# Long-Lived Ensembles of Shallow NV^–^ Centers in Flat and Nanostructured Diamonds by Photoconversion

**DOI:** 10.1021/acsami.1c09825

**Published:** 2021-09-01

**Authors:** Federico Gorrini, Carla Dorigoni, Domingo Olivares-Postigo, Rakshyakar Giri, Pietro Aprà, Federico Picollo, Angelo Bifone

**Affiliations:** † Istituto Italiano di Tecnologia, Center for Sustainable Future Technologies, via Livorno 60, 10144 Torino, Italy; ‡ Molecular Biology Center, University of Torino, via Nizza 52, 10126 Torino, Italy; § Istituto Italiano di Tecnologia, Center for Neuroscience and Cognitive System, corso Bettini 31, 38068 Rovereto (Tn), Italy; ∥ Department of Physics and “NIS Inter-departmental Centre”, University of Torino, Via Pietro Giuria, 1, 10125 Torino, Italy; ⊥ National Institute of Nuclear Physics, Section of Torino, Torino 10125, Italy; # Department of Molecular Biotechnology and Health Sciences, University of Torino, via Nizza 52, 10126 Torino, Italy

**Keywords:** diamond, nitrogen-vacancy centers, NV^0^, photoconversion, nanostructures, surface effects

## Abstract

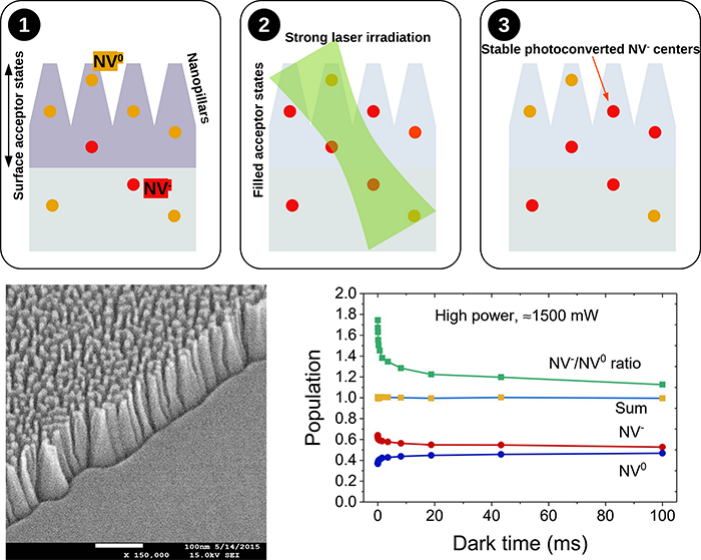

Shallow, negatively charged nitrogen-vacancy centers (NV^–^) in diamond have been proposed for high-sensitivity magnetometry and spin-polarization transfer applications. However, surface effects tend to favor and stabilize the less useful neutral form, the NV^0^ centers. Here, we report the effects of green laser irradiation on ensembles of nanometer-shallow NV centers in flat and nanostructured diamond surfaces as a function of laser power in a range not previously explored (up to 150 mW/μm^2^). Fluorescence spectroscopy, optically detected magnetic resonance (ODMR), and charge-photoconversion detection are applied to characterize the properties and dynamics of NV^–^ and NV^0^ centers. We demonstrate that high laser power strongly promotes photoconversion of NV^0^ to NV^–^ centers. Surprisingly, the excess NV^–^ population is stable over a timescale of 100 ms after switching off the laser, resulting in long-lived enrichment of shallow NV^–^. The beneficial effect of photoconversion is less marked in nanostructured samples. Our results are important to inform the design of samples and experimental procedures for applications relying on ensembles of shallow NV^–^ centers in diamond.

## Introduction

Negatively charged nitrogen-vacancy (NV^–^) centers are solid-state defects in the diamond lattice whose properties have been exploited to detect temperature gradients,^1,2^ magnetic^3,4^ and electric fields^5,6^ at the nanoscale, and interactions with magnetic molecules and nanoparticles.^7−10^ Due to their biocompatibility, NV^–^-enriched fluorescent nanodiamonds represent promising sensors to investigate the cellular microenvironment in living tissues and their use in high-sensitivity bioassays has been proposed.^11−13^ Furthermore, NV centers can be used in dynamic nuclear polarization (DNP) protocols where the polarization of the NV^–^s is transferred to ^13^C nuclei, leading to hyperpolarization of ^13^C nuclei in the diamond lattice.^14−16^ Substantial efforts are ongoing to promote polarization transfer from shallow NV centers to molecules absorbed at the diamond surface,^17^ thus enabling hyperpolarization of high-sensitivity tracers for biomedical magnetic resonance imaging.

For all these applications, the proximity of NV^–^ centers to the diamond surface, where NVs can effectively interact with spins outside the diamond lattice, is of paramount importance, as the coupling strength between magnetic dipoles decreases with increasing distance. To this end, specially engineered layers of shallow NV^–^s^18,19^ as well as nanodiamonds^20^ have been proposed.

Unfortunately, surface states and defects at and close to the diamond surface can affect the charge stability of NV centers, reducing the availability of magnetically active NV^–^ centers in favor of the neutral form (NV^0^ centers), which do not present the same detection features. The relative stability and interconversion between the neutral and negatively charged states of the NV centers have been the object of investigation in several studies,^21−25^ and various attempts have been made to increase the stability of NV^–^ centers, for instance, by surface termination,^26^ by doping of the diamond lattice,^27^ or by application of an electric field.^28^

Here, we investigate the effects of laser power and surface structures on charge stability and attainable spin polarization of shallow NV^–^ centers in high-purity diamonds. Specifically, we aim to establish experimental conditions that maximize the availability of magnetically active NV^–^ at the diamond surface. To this end, we apply fluorescence spectroscopy, as well as optically detected magnetic resonance (ODMR), in electronic-grade diamond samples implanted with nitrogen ions at different depths and with flat or nanostructured surfaces.

Importantly, we study the effects of these factors in a regime of high-power laser irradiation not previously explored, and we assess the time dependence of the laser-induced NV^–^/NV^0^ distribution during and after irradiation.

## Material and Methods

### Sample Fabrication

We used four electronic-grade, single-crystal diamond plates, with an initial nitrogen concentration lower than 5 ppb (Element Six Technologies Ltd). The samples were 2.0 × 2.0 mm^2^, 0.5 mm thick, with {100} face orientation, and they were double-side polished, with roughness *R*_a_ < 5 nm on both sides.

**Figure 1:**
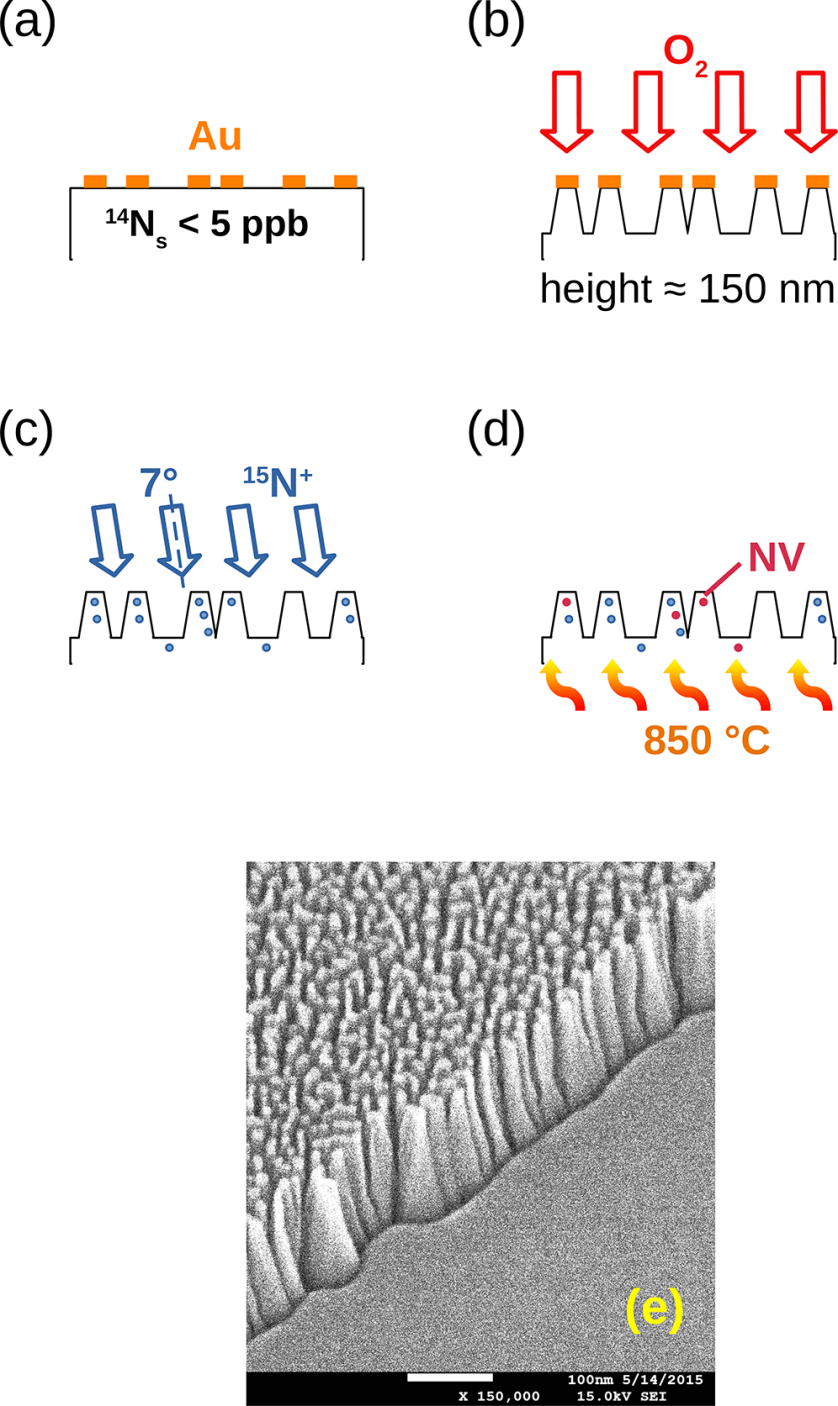
Nanofabrication process and synthesis of shallow NV centers in nanostructured samples. Electronic grade diamond with a very low concentration of nitrogen, less than 5 ppb, was masked with gold by sputtering (a) and then etched using oxygen reactive ion etching (b) to fabricate nanostructures, onto which nitrogen was implanted after gold removal (c). Samples were then annealed (d). SEM images show ≈150 nm high nanopillar-like structures (e). The SEM image was taken between steps (b) and (c), with still some conductive gold on the tips to improve image quality. Flat samples were implanted with ^15^N ions and then annealed, without application of any etching procedure.

Two of these samples were nanostructured to increase the surface-to-volume ratio. Samples nanostructuring was carried out using inductively coupled plasma-reactive ion etching (ICP-RIE) with oxygen gas, with a noncontinuous gold layer as a mask.^29^ Gold was deposited by DC sputtering (KS500 confocal sputtering system; Kenosistec Srl), using Ar gas (Figure 1a). Etching was performed by means of ICP-RIE (SI500—Sentech Instruments GmbH), using 700 W ICP power, 50 W plate power, and 40 sccm O_2_, at 1 Pa pressure, for 60 s (Figure 1b). After the ICP-RIE process, gold was removed with a commercial gold etchant (Sigma-Aldrich). Scanning electron microscopy (SEM) shows a tight arrangement of nanostructures (“nanopillars”) (Figure 1e) over the diamond surface, with a height of ≈150 nm. SEM images were acquired before gold removal.

Samples were implanted with ^15^N (INNOVION Corp., San Jose, CA) at a fluence of 10^13^ cm^–2^, at 7° angle from normal to avoid ion-channeling^30−32^ (Figure 1c), in two different energy conditions: one flat sample and one nanostructured sample were implanted at 10 keV; the other flat and nanostructured ones were implanted at 20 keV (see Table 1). Ion average ranges in the flat samples calculated by SRIM (http://www.srim.org, SRIM-2013.00) are, respectively, ≈15 nm, with straggling of 5 nm, and ≈27 nm with straggling of 8.5 nm.^33^

**Table 1:** Type of Surfaces and Implantation Energies of ^15^N^+^ for the Four Electronic-Grade Samples Used in This Work

sample name	surface type	implantation energy (keV)
F1	flat	20
F2	flat	10
N1	nanostructured	20
N2	nanostructured	10

We then annealed the four samples at a temperature of 850 °C for 2 h in high vacuum conditions (10^–6^ mbar) (Figure 1d). The setup employed consists of a vacuum chamber equipped with a dry pumping system to avoid hydrocarbon contamination and a resistive heater. The heating element is a tantalum box, 5 × 5 × 5 mm^3^ in size, that keeps the sample in thermal equilibrium with the surrounding blackbody radiation. The temperature was externally monitored through an optical window using a pyrometer, pointed to the tantalum box. The heating and cooling rates were of 10 °C/min to avoid thermal stresses in the diamond structures. At this temperature, vacancies and interstitial nitrogen atoms become mobile but substitutional nitrogen atoms are fixed in their position.^34^

We expect deeper NV centers for the 20 keV than for 10 keV implantation energies on average. For a rough estimate of the density of NV centers in the flat samples, we consider a conversion efficiency of ≈1% of the substitutional nitrogen after electron irradiation and annealing^35^ This corresponds to an NV areal density of 10^3^ μm^–2^. Dividing by the width of the implanted region (10 nm indicatively, the order of magnitude of nitrogen straggling), we find an NV density of 0.57 ppm. In the nanostructured samples, a more inhomogeneous distribution profile is expected, as the implantation occurs on an irregular profile.

The fabrication process for the nanostructured samples is summarized in Figure 1a–d. For flat samples, implantation and annealing procedures were the same as in the nanostructured samples but were performed on a flat surface.

### Experimental Setup

Full fluorescence spectra of NVs were taken with a confocal micro-Raman setup (LabRam Aramis, Jobin-Yvon Horiba), equipped with a 532 nm DPSS laser and an air-cooled multichannel CCD detector. Laser power was attenuated by neutral filters with 0, 1, or 2 optical densities (OD), resulting in laser intensities of 41 mW/μm^2^ (OD0), 4.1 mW/μm^2^ (OD1), and 0.41 mW/μm^2^ (OD2). When referring to full fluorescence spectra taken with the micro-Raman, we use interchangeably laser intensities or optical densities.

Time-resolved measurements were performed with a home-built fluorescence microscope equipped with a green 532 nm laser (5 W, Verdi, Coherent) and a 0.25 NA objective (Plan N, Olympus). A focal spot size of 10 μm^2^ was estimated. We used the same 532 nm laser to initialize and read out the state of the system. Fluorescence was collected by the objective and sent to a photon-counter module (Excelitas SPCM-AQRH-14-FC). Laser power at the sample surface was attenuated with a combination of absorptive filters and ranged from a maximum value of 1500 to 1 mW, depending on the experiment’s purpose. Correspondingly, the laser intensity could be tuned from a maximum of 150 mW/μm^2^ to 100 μW/μm^2^. For nanostructured samples, irradiation occurred on an irregular “conical frustum” surface, with flat tops and regions between the structures, and tilted side surfaces. As a result, laser power (and laser intensity) should be downscaled by a factor of 2.5–3. Even if this reduction in intensity may play a role, it cannot explain the large differences between flat and nanostructured samples reported in the Results section (differences are *as if* the laser power were 2 orders of magnitude less for the nanostructured samples). With the laser spot size calculated above, we estimated 10^4^ NVs to be simultaneously excited by the laser.

We collected fluorescence through two different spectral windows, selected by combinations of filters: 550 to 600 nm (“550–600 nm”) and >750 nm (“750 nm+”). The 550–600 nm window is centered around the zero-phonon-line (ZPL) of the NV^0^ centers, at 575 nm, and collects exclusively the NV^0^ signal. Conversely, the >750 nm window is more selective for the NV^–^ centers, even if a tail of the NV^0^ fluorescence can still leak through (see Section S1 of the Supporting Information).

We used a high-power acousto-optic modulator (ISOMET 523C-6) for pulsed-laser sequences. Typically, a variable preparation pulse was applied to initialize the system, followed by a read-out pulse. A read-out pulse of 5 μs provided good sensitivity while minimally perturbing the distribution of NV charge states even at the highest laser power used (see Section S2 of the Supporting Information).

The microwave lines were composed of a microwave generator (Keysight N5171B), an amplifier (ZHL-16W-43-S+), and a millimeter-sized gold-coated copper loop placed below the sample. A custom three-axis Helmholtz coil (Micro Magnetics, Inc) provided a static magnetic field along any desired direction. In some experiments, a strong magnetic field of 750 G was applied along the *z*-axis, perpendicularly to the top diamond face, to quench the spin dynamics and enable selective measurement of charge dynamics.^36^ The *z*-axis was set parallel to the [100] crystallographic direction so that the applied magnetic field had the same magnitude along the four possible orientations of the NV centers axes in the diamond lattice.

## Results

**Figure 2:**
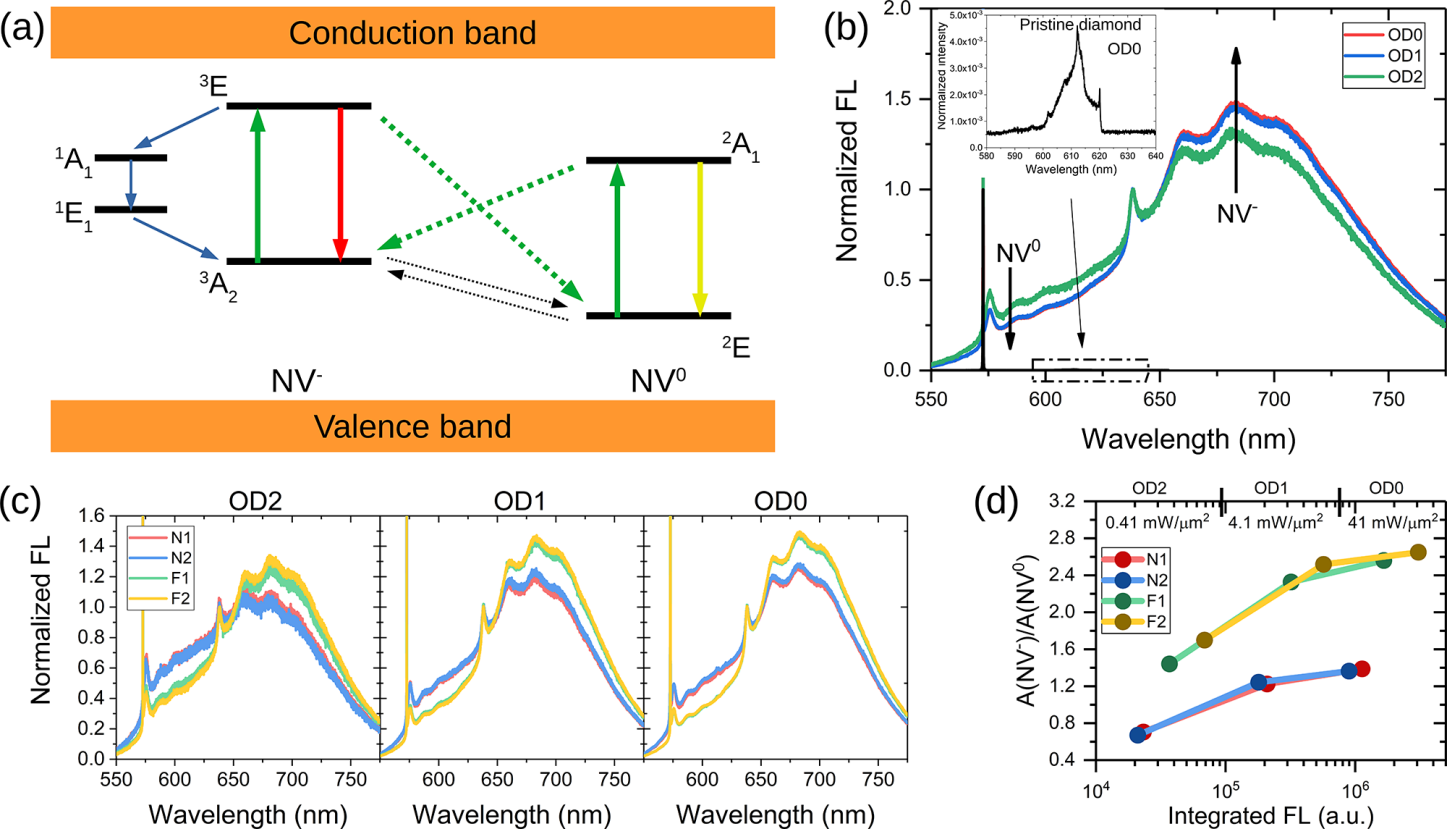
Fluorescence properties of NV centers. Electronic structures of NV^–^ and NV^0^ centers are represented in (a). Green arrows indicate optical excitation and photoconversion (continuous and dotted arrows, respectively). Red and yellow arrows denote radiative decay from NV^–^ and NV^0^ centers. Blue arrows indicate the nonradiative pathway, which leads to polarization of the *m*_s_ = 0 NV^–^ ground state. Black arrows represent tunneling transitions in the dark between the two charged states. (b) Both NV^–^ and NV^0^ fluorescence spectra were detected in implanted samples; no signal was detectable in nonimplanted, nonannealed samples (inset). Notably, emission from NV^–^s increases with laser power, at the expense of NV^0^s. (c): the FL of nanostructured samples is lower compared to flat samples at all laser power levels (indicated by the filter optical density (OD)), with a larger component of NV^0^. The ratio of NV^–^ and NV^0^ FL intensities is plotted in (d), as a function of total integrated fluorescence and laser intensity. The NV^–^/NV^0^ ratio increases with laser power in all samples and is systematically larger in flat samples.

Figure 2 shows the effects of laser power on the fluorescence (FL) spectra from different samples. The schematics of the energy level structures of NV^0^ and NV^–^ centers are reported in Figure 2a to facilitate the interpretation of the experimental results. In short, NVs are characterized by a ground state (a spin-triplet ^3^A_2_ for NV^–^, and ^2^E for NV^0^) and excited states (^3^E for NV^–^ and ^2^A_1_ for NV^0^) positioned within the diamond band gap,^37^ each accompanied by a phonon band. Additionally, the NV^–^ has two intermediate states (^1^A_1_ and ^1^E) coupled to the ground and the excited states. The existence of this coupling enables a nonradiative transition that accumulates population in the *m*_s_ = 0 levels of the ground state under constant laser irradiation (blue arrows). A green laser (532 nm) is used to excite both the NV^–^ and the NV^0^ centers (green arrows). Laser irradiation can also induce a charge-state conversion, either by promoting an electron from the NV^–^ excited state into the conduction band (NV^–^ → NV^0^ conversion) or by exciting an electron from the valence band to the NV^0^ ground state (NV^0^ → NV^–^ conversion).^38^ These two routes of photoconversion are represented by green dotted arrows. Experimental observations suggest that, upon switching off the laser, the system goes back to equilibrium through charge conversion in the dark, a process attributed to electron tunneling (black dotted arrows). Indeed, tunneling couples the NV centers to proximal electron donor or acceptor states, such as substitutional nitrogen atoms, vacancy complexes, and surface states.^23,25,39^

Figure 2b shows fluorescence spectra from one sample (F2) at different laser powers. The NV charge states are characterized by different fluorescence spectra, with zero-phonon lines (ZPL) at 575 and 638 nm for the NV^0^ and NV^–^, respectively, and a phonon sideband peaked around 620 nm for the NV^0^ and 700 nm for the NV^–^, and extending up to ≈800 nm in both cases. Spectral features from both NV^–^ and NV^0^ centers are apparent, demonstrating the co-occurrence of the two charge states in this sample. The NV centers originate exclusively from implanted nitrogen (pristine diamond samples before implantation and annealing show no detectable fluorescence under a maximum laser intensity excitation of 41 mW/μm^2^ but only the first- and second-order Raman peaks, shown in the inset). As the laser power intensity increases by 2 orders of magnitude (from 0.41 mW/μm^2^ for filter optical density OD = 2 to 41 mW/μm^2^ at OD = 0), the relative intensity of the NV^–^ band increases with respect to that of the NV^0^ band. This indicates some degree of NV^0^ → NV^–^ photoconversion at higher laser powers under continuous excitation (curves are normalized to 1 at 638 nm). The spectra in Figure 2c reflect the relative abundances of NV^0^ and NV^–^ centers in the nanostructured and flat samples for different laser powers. For the two nanostructured samples, the overall FL is lower (not apparent in the figure, where spectra are normalized for comparison), and the FL spectra show a higher component in the NV^0^ region (blue and red curves compared to yellow and green curves). In all samples, an increase in the NV^–^ component and a decrease in NV^0^ is observed with increasing laser power. We summarize these observations in Figure 2d, where the *x*-axis represents the integrated total FL, comprising the signals from both NV types, while the *y*-axis indicates the ratio of the areas under the NV^–^ and NV^0^ curves. Fluorescence curves were deconvolved into NV^0^ and NV^–^ components before calculating the values of Figure 2d, similar to that in Alsid et al.^40^ (the process of integration and calculating the FL ratio is described in Section S1 of the Supporting Information). For all samples, the NV^–^/NV^0^ ratio increases with laser power, suggesting stronger NV^0^ → NV^–^ photoconversion at higher laser powers. Nanostructured samples (N1 and N2) contain fewer NV centers (reflected by an overall lower FL, integrated over the entire spectrum) and a higher fraction of NV^0^ compared to flat samples.

**Figure 3:**
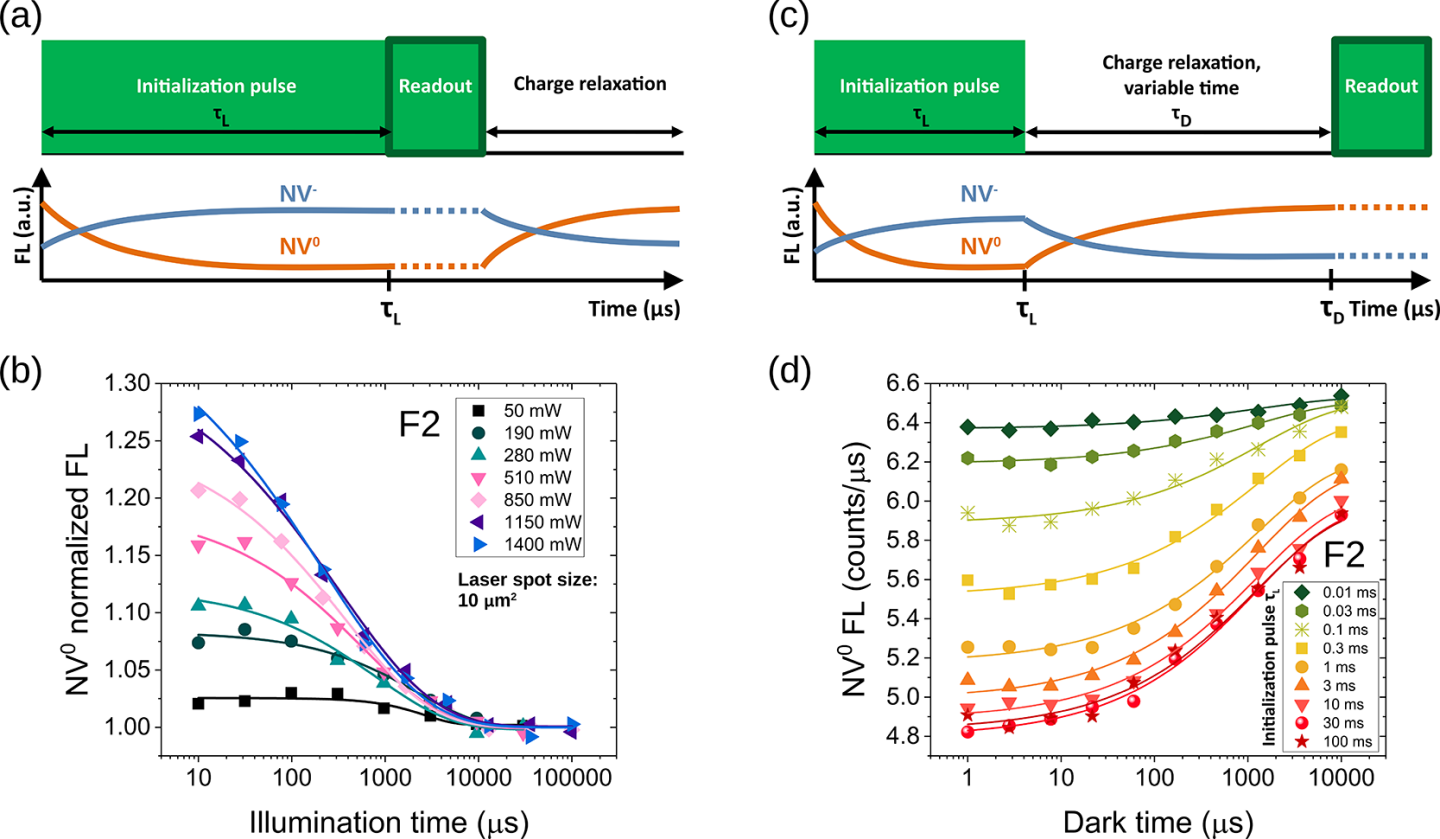
Detection of charge dynamics. (a) Pulse sequence adopted to investigate charge dynamics under laser pumping, for variable irradiation pulse length τ_L_. Laser-induced NV^0^ → NV^–^ photoconversion results in a decrease of the NV^0^ signal and an increase of the NV^–^. As an example, panel (b) shows the decrease in NV^0^ FL for sample F2, as measured in the 550–600 nm window, for different laser powers. (c) Pulse sequence used to detect the charge recovery in the dark after a preparation pulse. Recovery of NV^0^ FL for sample F2 at a laser power of 625 mW is shown in panel (d). Various curves in panel (d) correspond to different durations of the initialization pulse.

To explore the dynamics of laser-induced charge switching and subsequent return to equilibrium, we applied two pulsed experimental schemes (Figure 3). In these experiments, a strong magnetic field (750 G) was applied along the [100] direction to suppress the effects of spin polarization of FL and selectively investigate charge dynamics. In fact, a sufficiently strong magnetic field (>600 G) quenches the polarization of the *m*_s_ = 0 ground state by spin-state mixing and leads to a reduction of the spin-related component in the FL.^36,41,42^

Figure 3a,c shows the two pulse sequences adopted to assess charge dynamics during laser irradiation and in the following dark time. In the first one (Figure 3a), a laser pulse (532 nm) of variable duration τ_L_ (from 10 μs to 100 ms) was focused on the diamond surface to study charge photoconversion during irradiation. This pulse induces photoionization, with an increase in NV^–^ and a decrease in the NV^0^ signal (blue and orange curves, respectively). FL was recorded by the subsequent short detection pulse of 5 μs. At the end of the sequence, a time interval of 10 ms, during which the laser was off, allowed for charge relaxation before the experiment was repeated for signal averaging. Data points acquired after discrete initialization pulses, for different values of laser power, are shown in Figure 3b (for sample F2). For simplicity, only FL signals acquired in the range 550–600 nm, reflecting predominantly NV^0^ fluorescence, are reported (the behavior of NV^–^ is symmetrical). The curves reported in this figure show that the degree of photoconversion increases steeply with lasers power, ranging between 50 mW to 1400 mW. Independently of laser power, for pulses longer than 10 ms, the fluorescence level lies within 5% of its equilibrium value, and photoconversion approaches saturation in all samples. Therefore, in the following experiments, we use an initialization pulse of at least 10 ms to maximize the number of NV^–^.

The second pulse sequence of Figure 3c was used to probe the charge dynamics in the dark. The sequence consists of a laser pulse of duration τ_L_ followed by a variable dark time τ_D_ (from 1 μs to 10 ms) and a 5 μs readout pulse. In Figure 3d, the FL of the NV^0^ centers measured through the 550–600 nm spectral window is reported as a function of τ_D_. As discussed above, the first initialization pulse reduces the number of NV^0^ by photoconversion to NV^–^. During the dark time, the system goes back to equilibrium and a steady increase in the number of NV^0^ centers is reflected by increased fluorescence in the spectral region 550–600 nm registered by the readout pulse. A plausible explanation of this recharging-in-the-dark process, previously reported and discussed,^23,25^ contemplates electrons tunneling from the negative NV^–^ to surface acceptors or vacancies until equilibrium is reached. To exemplify this phenomenon, Figure 3d shows the curves obtained with a laser power of ≈625 mW and initialization times from 10 μs to 100 ms for sample F2. Coherent with the results of Figure 3b, the initial value of fluorescence decreases with longer initialization pulses, meaning that an increasing fraction of NV^0^ is converted into NV^–^ upon laser irradiation. In all cases, fluorescence from NV^0^ increases in the dark with longer τ_D_. We also notice that the curves acquired after a τ_L_ of 10, 30, and 100 ms are almost identical, consistent with the observation that beyond 10 ms the photoconversion process saturates.

The curves of Figure 3b can be fitted with a stretched exponential law of the type^8^
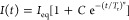
1where *T*_r_ represents a characteristic timescale for the photoconversion process under the laser pulse, *n* is the stretching factor, *I*_eq_ is the FL value at equilibrium, and *C* is a positive parameter describing the fluorescence drop from the initial value to equilibrium. Curves were normalized by *I*_eq_ for easier comparison. Charge recovery curves, as in Figure 3d, can also be fitted with the function of eq 1, even though, in this case, the pre-exponential parameter is negative (the initial value is lower than the equilibrium value). It should be noted that the mechanisms underlying charge conversion in the dark are different from those that induce photoconversion, so the two characteristic times *T*_r_ are not related.

**Figure 4:**
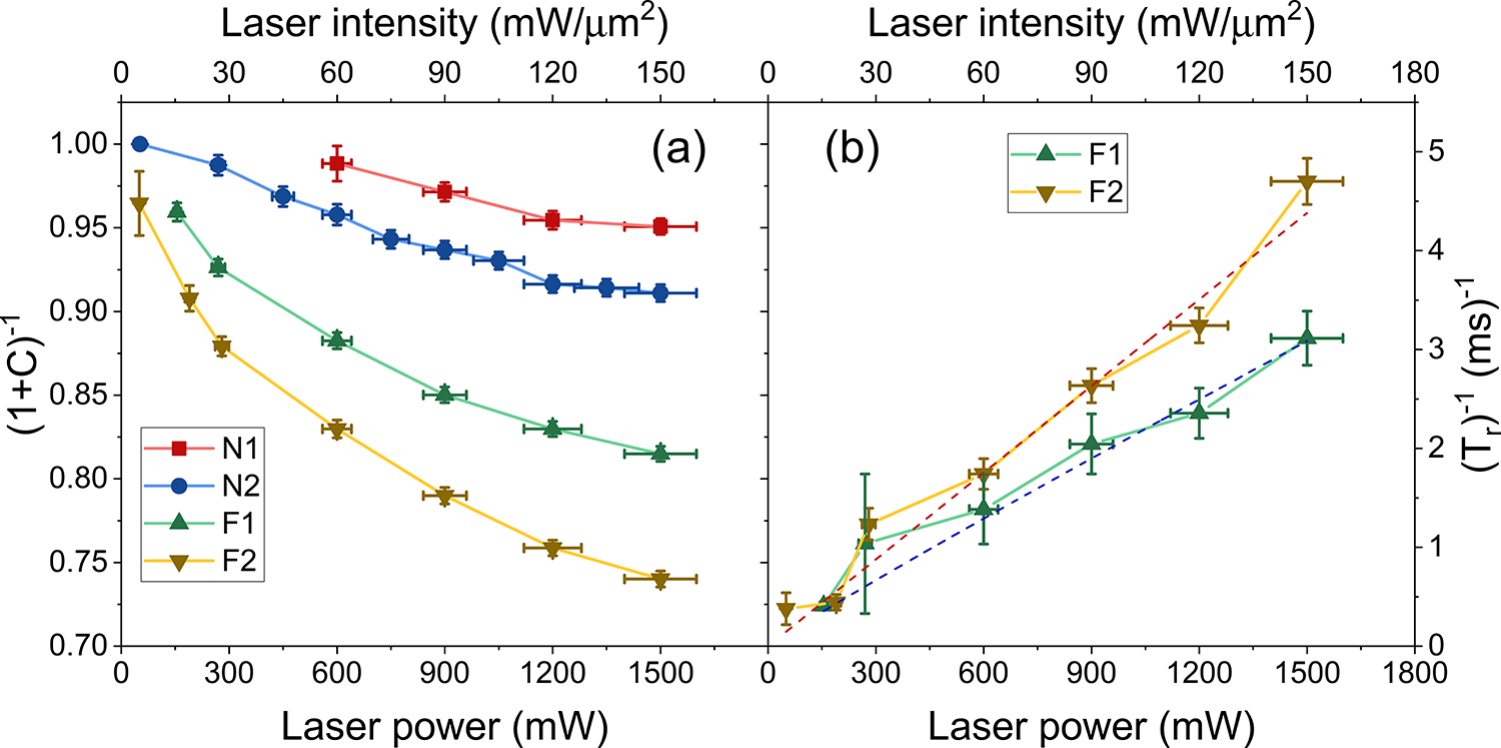
Parameters describing NV^0^ → NV^–^ photoconversion under laser irradiation. The quantity (1 + *C*)^−1^ in (a) indicates the ratio between the equilibrium value of FL and the initial value, right after the initialization pulse. The decrement is more consistent for the flat samples (down to 75% for sample F2). The photoconversion rate (*T*_r_)^−1^ displayed in (b) is nearly linear with laser power, following a power law with exponents of 0.95 ± 0.07 for F1 and 1 ± 0.07 for F2 (red and blue dashed curves).

The photoconversion data for all samples are reported in Figure 4a, where we plot (1 + *C*)^−1^ (eq 1), corresponding to the ratio between the FL equilibrium and initial values, for different laser powers. The largest drop in NV^0^s due to photoconversion was observed in the flat samples (down to 74% for F2 and 81% for F1) at the largest available laser power, while more limited variation was observed in the nanostructured samples. Note that the large error bars on the *x*-axis are caused by a slight deterioration in the laser beam’s transmittance at high laser power, an effect that accounts for 5–10% of losses during prolonged irradiation. In Figure 4b, we show the rate of photoconversion (i.e., the inverse of the photoconversion time *T*_r_) for the flat samples (in the case of nanostructured samples, the uncertainties on extracted values are too large to draw conclusions on their laser-power dependence). (*T*_r_)^−1^ is seen to increase with laser power according to a power law with exponents of 0.95 ± 0.07 and 1 ± 0.07 for samples F1 and F2, respectively (dashed lines). The nearly linear dependence on the laser power is suggestive of a single-photon-mediated NV^0^ → NV^–^ photoconversion mechanism.

**Figure 5:**
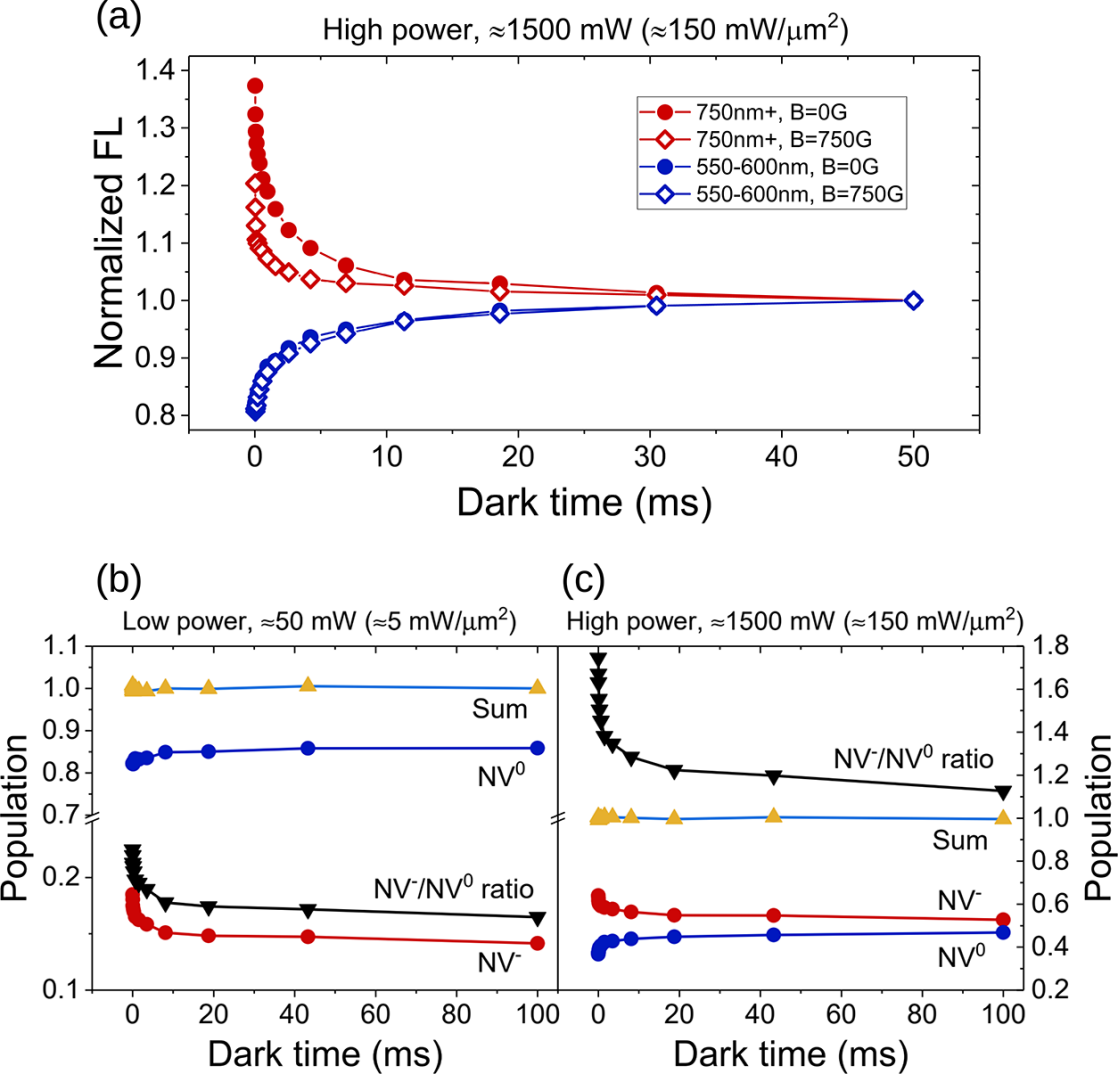
Spin and charge dynamics after the laser pulse. The effect of a strong magnetic field (750 G) on the fluorescence of sample F2 at a laser power of 1500 mW is shown in (a). FL was collected in the 550–600 nm region (blue curves) and in the >750 nm window (red curves), with and without magnetic field (empty and solid symbols, respectively). When considering only the NV^0^ centers (550–600 nm window), no change in the FL profile is observed, irrespective of the magnetic field applied. On the contrary, the signal in the >750 nm window is reduced by a strong magnetic field. This difference is the result of quenching NV^–^ spin polarization through spin state mixing. However, beyond ≈10 ms, the difference vanishes, and the evolution at longer times is dominated by charge dynamics. At low (b) and high (c) laser powers, the 10 ms laser pulse creates an excess of NV^–^s (red curve) that convert to NV^0^s (blue curve) in the dark. NV^–^ and NV^0^ populations are normalized such that their sum is 1 (yellow-azure curve). The ratio *R* is indicated by the black curve. A near-equilibrium ratio (0.15) is reached after 100 ms at low power (50 mW), while a large and sustained *R* (1.13) value is observed after high power irradiation (1500 mW). Results of (b) and (c) are taken at a magnetic field of 750 G, which enables determining the charge state ratio (for more details, see the text or Section S4 of the Supporting Information).

To illustrate the symmetry between the dynamics of the two charge states in the dark, in Figure 5a, we show the FL signal in the 550–600 and >750 nm spectral windows, dominated by NV^0^ and NV^–^, respectively, for sample F2 at the highest laser power. The experiment of Figure 3c was performed with or without an applied magnetic field of 750 G (open and solid symbols in Figure 5a, respectively). Indeed, while the evolution of the NV^0^ signal reflects strictly charge dynamics, the NV^–^ signal is influenced by both charge and spin dynamics. The applied magnetic field suppresses spin dynamics and makes it possible to selectively measure the effects of charge switching for NV^–^. Under these experimental conditions, the two curves, in red and blue, representing charge state recovery for NV^–^ and NV^0^, appear symmetrical, thus corroborating the idea that laser light converts one state into the other.

Performing the experiment of Figure 3c with the two 550–600 and >750 nm spectral windows makes it possible to estimate the charge-state ratio *R* = [NV^–^]/[NV^0^] at a steady state, as proposed by Giri et al.^36^ (derivation of *R* is illustrated in Section S4 of the Supporting Information). The two assumptions are (i) all the NV centers are optically active and (ii) the sum of NV^–^ and NV^0^ is always constant. The latter hypothesis implies that a reduction of NV^0^ is counterbalanced by an increase of the NV^–^, and vice versa. Figure 5b,c shows the subdivision of the populations between NV^–^ and NV^0^ (at *B* = 750 G) after a 10 ms laser pulse at low and high powers for sample F2, as an example. In both cases, the initialization pulse produces an excess of NV^–^ centers that convert back into NV^0^ during the dark time. At low laser power (50 mW, ≈5 mW/μm^2^), the signal is dominated by NV^0^s, with a ratio *R* = 0.13/0.87 ≈ 0.15 at the longest time point acquired (100 ms). However, at high power (1500 mW, corresponding to ≈150 mW/μm^2^) the ratio changes to 0.53/0.47 ≈ 1.13, with the NV^–^ centers now being the dominant state after a sevenfold increase compared to the low power case. Interestingly, after an initial rapid decrease, a large value of *R* (1.13) is observed after 100 ms, and the curve exhibits a much more slowly decaying component. Thus, the effect of an intense laser pulse is to create a large NV^–^/NV^0^ ratio that is sustained over tens or hundreds of milliseconds (see the Discussion section). Evidently, the charge dynamics has fast and slow components, summarized by the stretched exponential behavior, with the fast components in the 10–1000 μs timescale and the slower ones on a 100 ms scale (Figure 5b,c). On the contrary, the timescale of spin dynamics is of the order of ≈1 ms. For this reason, when considering the combined effect of spin and charge dynamics, there will be a substantial overlap in the fluorescence up to 1–10 ms, while at longer times the FL profile will be dominated by charge dynamics. This is also apparent from Figure 5a, where the difference between the two red curves, attributed to spin dynamics, vanishes after ≈10 ms; the evolution at longer times is unaffected by the magnetic field, thus indicating that it can be attributed exclusively to charge dynamics.

Finally, repeating the same experiments with sample N2 gives similar results, but with a modest degree of NV^0^ → NV^–^ photoconversion and a shorter lifetime of the NV^–^-photoconverted centers (approximately 10 ms). Even at the highest power of 1500 mW, the ratio NV^–^/NV^0^ is 0.1/0.9 ≈ 0.11, much lower compared to the results of sample F2 (as shown in Figure S4 in Section S4).

Finally, we report optically detected magnetic resonance (ODMR) experiments to assess the spin polarization of the ground-state level *m*_s_ = 0 of NV^–^. Continuous laser irradiation polarizes the *m*_s_ = 0 state, while a sweeping microwave field transfers the population to the *m*_s_ = ± 1 state. However, we note that the detected ODMR contrast is also affected by NV^0^ centers. Indeed, even though FL is collected in the >750 nm window, where most of the signal comes from the NV^–^ centers, a residual background signal from NV^0^s is still present. This residual NV^0^ signal is not modulated by microwave irradiation and cannot be removed entirely by spectral filtering. Hence, we expect the ODMR contrast to be increased at higher laser power due to two independent mechanisms: (i) increased NV^–^ polarization and (ii) reduction in the nuisance background signal from NV^0^.

**Figure 6:**
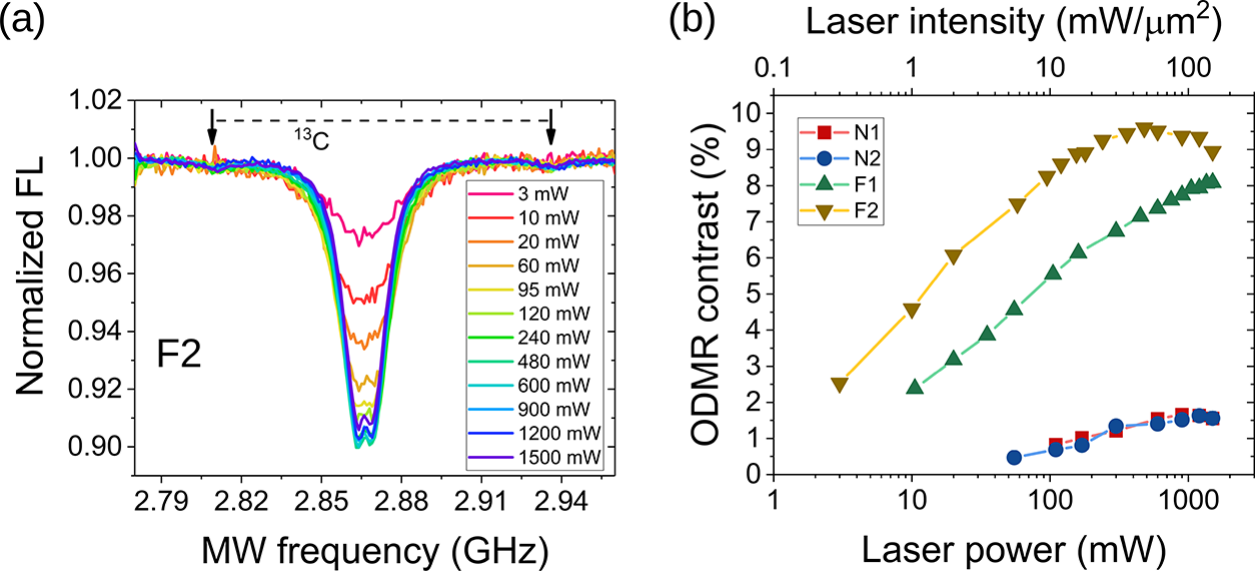
Optically detected magnetic resonance. (a) ODMR spectra from sample F2 as a function of laser power. (b) ODMR contrast for all samples. The increase, in contrast, is more pronounced for flat samples.

In Figure 6a, we show the ODMR spectra from sample F2 for a fixed microwave power and increasing the laser power up to 1500 mW. The contrast (i.e., the depth of the dip) increases rapidly with laser power and makes it possible to resolve the strain-split doublet^43^ and the ^13^C sidebands at the highest contrast-to-noise ratio. In Figure 6b, we show the dependence of the ODMR contrast on laser power for all samples. Nanostructured samples exhibit low contrast and limited gain with increasing laser power, consistent with the evidence that NV^0^ remains the dominant charge state even at the highest laser powers. The ODMR contrast in flat samples is much larger and increases sharply with laser power. Saturation of contrast for the F2 sample at around 600 mW probably reflects laser-induced repolarization related to insufficient microwave power (see the Discussion section). In light of the above results, we can conclude that the increment in the ODMR contrast with laser power does not merely reflect increased ground-state spin polarization, but also a gain in the number density of NV^–^ as a result of photoconversion (see the Discussion section for the consideration on the ODMR contrast).

## Discussion

Our results highlight the importance of NV^0^ to NV^–^ charge conversion in shallow defects, and point to the use of high-power laser irradiation to improve the availability and polarization of shallow NV^–^. This effect appeared to be more prominent in flat samples, compared to nanostructured samples.

The maximum values of laser intensities used here are larger than those commonly reported in the literature. In fact, NV centers are typically investigated using laser intensities of few mW/μm^2^ or less^21,44,45^ and only rarely approach 100 mW/μm^2^.^40^ The use of such a high laser power is justified by the lack of saturation of the NV optical transition, as shown in Figure S3 (Section S3 of the Supporting Information). Indeed, we recorded FL emission in the two windows “550–600 nm” and “>750 nm” with increasing laser power: the signals from the >750 nm window barely show any indication of saturation (saturation power estimated at 8.5 W), while the 550–600 nm window shows a flexion only for the flat samples (saturation power estimated at 2.3 W). Thus, we can conclude that the maximum laser power used here was below the onset of saturation. These results are not surprising, as saturation curves depend on whether a single NV center or ensembles are observed and on the sample characteristics (defects type and density). Very different saturation intensities are reported in the literature, ranging from a few mW/μm^2^^46−48^ to an estimate of thousands of mW/μm^2^.^49^

In all four samples investigated, the NV^0^ centers represent a substantial proportion of NV defects. In the bulk, the presence of electron donors, such as substitutional nitrogen, favors the formation and stability of the NV^–^ center. However, moieties and acceptor states at the diamond surface decrease the stability of the NV^–^, even at depths of tens of nanometers,^50−52^ and NV^0^s may become the dominant charge state^53,54^ with detrimental effects for sensing and polarization transfer applications. Our samples were engineered to present shallow NV centers (similar to the previous reports^55^), and it is not surprising to find predominantly NV^0^ centers. In the case of nanostructured samples, the surface effects are exacerbated by the large surface-to-volume ratio of the nanostructures. Therefore, diamonds with nanopillars have fewer NV^–^s compared to NV^0^s, as apparent in the FL and ODMR spectra (Figures 2c, 4a, and 6b, respectively).

Moreover, the integrated fluorescence of Figure 2d suggests that nanostructured samples have a lower number of NV centers altogether, independent of the specific charge state. This could be determined by less efficient ion implantation on the nanostructures or by a loss of nitrogen due to ion straggling.

Laser irradiation has two effects on the NV centers. The first effect is the polarization of the NV^–^*m*_s_ = 0 spin state. The second effect is to convert part of the NV^0^s into NV^–^ and increase the ratio of the negatively charged centers over the total NVs. Upon switching the laser off, a process of recharging in the dark results in an NV^–^ → NV^0^ back-conversion. An interesting finding of this work is that the excess NV^–^ population is persistent over a relatively long timescale.

The following hypothesis can qualitatively explain the experimental findings. A laser pulse ionizes the substitutional nitrogen atoms,^56^ and electrons are released into the conduction band and recombine with electron-trapping defects,^57^ including the NV^0^. The decrease of NV^0^ FL in Figure 3b is consistent with this mechanism. The phenomenon of redistribution of charges was recently investigated from a different perspective, namely the Stark shift of optical transition.^39^ The nearly-linear dependence of (*T*_r_)^−1^ on laser power (Figure 4b) suggests a single-photon ionization process of substitutional nitrogen as a source of electrons for NV^0^ → NV^–^ photoconversion. We note that the proposed mechanism for NV^0^ → NV^–^ charge-state conversion is different from the two-photon excitation of electrons from the valence band (i.e., a photon initializing the NV^0^ to the excited state and a second photon promoting an electron from the valence band to a trapping NV^0^ centers),^38^ which is better described by a quadratic dependence of (*T*_r_)^−1^ on laser power.^21^ In the dark, an NV^–^ → NV^0^ back-conversion is observed. The mechanism proposed for this process is electron tunneling, which critically depends on the NV environment. Tunneling to a neighboring trap can only occur if the trap is empty.^25^ At weak laser powers, the photoexcited electrons do not saturate the traps in the vicinity of the NV center. At stronger laser powers, increasingly distant traps are populated. Hence, the time the system takes to return to equilibrium depends on laser power, as tunneling to distant traps is a much slower process. This picture is consistent with the results of Figure 5b,c that show, at high laser power, a large [NV^–^]/[NV^0^] ratio persistent on a scale of 100 ms. Thus, high-power irradiation increases the availability of NV^–^ and extends the time window in which the NV^–^ spin states can be manipulated and detected, with important implications for sensing and polarization-transfer applications.

The beneficial effects of high-power irradiation are less pronounced in the etched samples, with a modest increase in NV^–^ population. This is consistent with the idea that surface defects compete with NV^0^ to capture released electrons. Therefore, the NV^0^ → NV^–^ conversion is more efficient in the flat samples and less efficient in the nanostructured ones, where the amount of surface defects is large compared to the amount of NVs (Figure 4a). In our samples, the advantage of the increased exposed area resulting from nanostructuring appears to be mitigated by the less favorable charge dynamics. Additional treatment of the surface to stabilize NV^–^ (like selective oxidation^58^ or fluorination^26^) may improve the performance of nanostructured samples, but we have not explored the effects of different terminations in this paper. It is interesting to compare our results concerning nanopillars with recent work,^59^ where nanostructures demonstrated higher brightness, larger NV^–^/NV^0^ ratio, and better spin properties compared to a bulk reference. We can explain the differences by accounting for the striking different sizes of nanostructures. In the work of McCloskey,^59^ the nanopillars size was ≈1 μm (as an order of magnitude for both height and width), while our nanostructures were much smaller, with a height of 150 nm and a width narrowing from 50 nm at the base to 10 nm at the top. Therefore, the effects of lateral surfaces are expected to be relevant for small nanostructures but negligible for micrometric pillars, where only a small fraction of the NV centers (the closest to the sidewall) is affected. Additional work would be necessary to trace the transition from nano- to microstructures in terms of NV^–^/NV^0^ ratio and to understand if the size and shape can affect the way photoexcited electrons recombine with defects, including NV^0^.

Interestingly, both routes of photoconversion (NV^–^ → NV^0^ and NV^0^ → NV^–^) have been reported in the literature (see, for instance, refs (40, 44, 57, 60) for NV^–^ → NV^0^ and refs (25, 55) for NV^0^ → NV^–^). These different trends of charge-state conversion are related to the environment of the NV centers, i.e., to the presence of electron donors (mostly substitutional nitrogen) and electron acceptors (like vacancies created by nitrogen implantation or surface acceptors). Thus, the photostability and the routes of photoconversion are in general different for bulk and shallow NV centers, as suggested by the recent literature.^36,54^ Our results apply to shallow NV centers, which are relevant for sensing and polarization-transfer applications. Importantly, we stress the fact that in our samples a high laser power has a twofold beneficial effect: first, it increases the density of NV^–^s by photoconverting part of the NV^0^s, and second, it polarizes the NV^–^ spins. This might not be the case for bulk samples, as improved spin polarization at high laser power could be frustrated by a loss of NV^–^ centers via NV^–^ → NV^0^ photoionization.

An intermediate scenario between shallow NV centers and native bulk NV centers is represented by high-energy nitrogen implantation (hundreds of keV or MeV). On the one hand, it has been reported that, under these conditions, the NV yield from nitrogen is extremely high (approximately 50% for 2 MeV^61^ and 18 MeV^35^ of implantation energy), so a high density of NV centers can be generated at a depth of 1 μm or more, where the effects of surface acceptors on the NV^–^ stability are negligible. On the other hand, high implantation energies and doses are accompanied by the creation of a large number of vacancies, divacancies, and vacancy clusters, which might deteriorate the diamond lattice and compromise the NV^–^ stability.^62,63^ The effect of nitrogen implantation energy and dose on NV^–^ and NV^0^ charge dynamics might be the subject of a future analysis.

We used stretched exponentials to fit all the fluorescence curves to account for heterogeneity in the ensemble of NVs, resulting in a superposition of many single exponential curves.^8,64^ The dynamics of charge tunneling was addressed by Miller^65^ and Tachiya^66^ by considering a spatially homogeneous distribution of charge acceptors and recently extended to a discrete distribution.^23^ In this model, the charge dynamics can be described by a functional dependence of the form
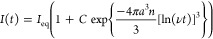
2Here, *n* is the density of trapping defects, and ν and *a* are parameters linked to the NV^–^ center, defined as the tunneling frequency and the attenuation length of the wavefunction, respectively. Using this expression for the fits, we find *a* = 0.1 nm, ν = 0.8 ± 0.1 MHz, and *n* = 2–4 ppm. The density *n* shows a slight tendency to decrease after long laser pulses, possibly indicating that photoexcited electrons have filled some charge traps. Additional work will be necessary to understand the features of *n* and some subtleties not captured by the present model, such as nonuniform values of *n*, ν, and *a* across a defective sample. However, for our data, we do not observe a substantial improvement of the fit using eq 2 compared with a stretched exponential function (eq 1). We are mostly interested in the initial out-of-equilibrium values of the curves compared to equilibrium. These values are set during the initialization phase by the laser pulse length and power and do not depend critically on the choice of the fitting function. In this respect, the values returned by the two fitting procedures are in good agreement.

The leakage of the NV^0^ FL component in the NV^–^ spectral region can affect the ODMR contrast.^67−69^ For NV^–^ centers, the contrast is defined as *C*_s_ = (*I*_off_ – *I*_on_)/*I*_off_, where *I*_off_ and *I*_on_ are the collected FL intensities with microwaves off- and on-resonance, respectively. If the intensity contains an NV^0^-related component *I*_0_, then the contrast *C*_s_ must be scaled by a factor *I*_off_/(*I*_off_ + *I*_0_). In our case, the intensity *I*_off_ (*I*_0_) is the fraction *f*^–^ (*f*^0^) of the total NV^–^ (NV^0^) intensity filtered by the >750 nm longpass and normalized by photon-counter efficiency. It is possible to relate these intensities to the charge-state ratio *R* by parameters pertaining to NV^–^ (NV^0^) centers, such as the absorption cross sections σ^–^ (σ^0^), and the excited-state lifetime τ^–^ (τ^0^). The ODMR contrast for the NV ensemble is
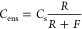
3where  is a numerical factor that depends on detection conditions, such as the choice of filters and detectors, and structural properties of NV centers. Conversely, *R* can vary for different samples. For shallow NVs, where the charge state is dominated by neutral centers, *R* is small and the ensemble contrast is substantially reduced. On the contrary, for samples that mostly contain NV^–^ centers (as seen usually for bulk NV centers), *R* is much larger than *F* and the correction is minimal. For our setup, we estimate *f*^0^/*f*^–^ ≈ 0.42 from the shape of fluorescence (see Section S1). Using σ^–^ = 3.1 × 10^–17^ cm^2^,^70^ σ^0^ = 1.8 × 10^–17^ cm^2^,^71^ τ^–^ = 12 ns, and τ^0^ = 21 ns,^72^ we find *F* ≈ 0.14. Also, the charge state ratio varies dramatically from 0.15 at low laser power to 1.13 at high power (Figure 5b,c). With an indicative value of *C*_s_ = 10%, we estimate ensemble contrasts of 5.2 and 8.9% at low and high laser powers, respectively, and a tendency to increase with *R* and with laser power, following the NV^0^ → NV^–^ photoconversion. In principle, *C*_s_ can be recovered *a posteriori* from experimental values of the ODMR contrast, provided that the dependence of *R* on the laser power is known. The proposed interpretation explains the trend shown in Figure 6b from low to moderately high laser powers, although the contrast shows a saturation or even a slight reduction at the highest power levels (see, for instance, sample F2). It also explains the striking difference between the flat and the nanostructured samples: in the latter, NV^0^s are by far the dominating species even at high laser power and the extracted contrast is always poorer than in the flat samples. Concerning the saturation of the contrast at high powers, we note that an optimal contrast results from combined laser and microwave spin initialization. If the laser power is too high, the microwave field cannot compete in inverting the population levels in the ground state, and some degree of laser-induced repolarization is expected.^49^ Despite setting the power of the microwave to a maximum available value (output of 8 dBm amplified by ≈35 dB), the laser repolarization effect may prevail, resulting in the saturation and slight reduction shown in Figure 6b.

The resulting increase in ODMR contrast improves sensitivity η (expressed in ) and the minimum magnetic field δ*B* detectable at time Δ*t*, defined as . In continuous-wave ODMR protocols, sensitivity is given by the relation^73^
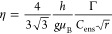
4where the numerical factor in front is related to the Lorentzian function used to fit the resonance, *h* is the Planck constant, μ_B_ is the Bohr magneton, *g* ≈ 2, Γ is the full width at half-maximum of the Lorentzian, and *r* is the photodetection rate. The contrast *C*_ens_ in eq 4 is the ODMR contrast of the NV ensemble given by eq 3. At low laser power (50 mW), we find experimentally *r* = 0.73 × 10^6^ s^–1^, while at high power (1500 mW), we find *r* = 17.8 × 10^6^ s^–1^ (values normalized by limited photon counter efficiency of the detector). Taking values of *C*_ens_ and *R* as before, and a representative Γ = 10 MHz, we find  and  at low and high powers, respectively. Sensitivity might be further improved with the use of pulsed-wave ODMR^73^ and extended to the detection of AC magnetic fields.^74,75^ Therefore, magnetometry applications benefit from the use of strong laser power by an increase in the photon detection rate and improved contrast. We note that the correction factor of eq 3 does not exclusively determine the ODMR contrast but plays a role in other detection schemes, including pulsed sequences as those in Figure 3a,c.

^13^C nuclear hyperpolarization is another technique that could benefit from an increased fraction of NV^–^s. This technique is based on the transfer of spin polarization from highly polarized NV^–^ to first-neighbor ^13^C nuclei via spin–spin interaction at specific values of the applied magnetic field.^76^ Polarization can then diffuse through the diamond lattice to the bulk ^13^C spin reservoir by nuclear spin–spin interactions.^77,78^ However, this process is much slower compared to the fast hyperpolarization of the first shell ^13^C nuclei (≈1 ms) in the vicinity of NV^–^ centers, and increasing the amount of NV^–^s via photoconversion can substantially improve the efficiency of the hyperpolarization process.

The effects reported here may be particularly important for hyperpolarization of nuclei outside the diamond surface. Indeed, it has been proposed that shallow NV^–^ may provide a source of spin polarization of molecular moieties adsorbed at the diamond surface,^16,17,79^ thus complementing techniques like dynamic nuclear polarization or optical pumping spin-exchange. Increasing their concentration in the vicinity, the diamond surface may enable hyperpolarization of nuclei outside the diamond lattice.

Realistically, laser power is constrained by the available equipment and by the nature of the sample. Some applications of sensing,^80^ widefield imaging,^81^ and hyperpolarization^17^ necessarily address a large number of NVs over broad two-dimensional (2D) or three-dimensional (3D) regions at the expense of a tighter focusing (so, with lower laser intensity). Nevertheless, our results indicate that charge-state photoconversion must be taken into account for shallow NV centers and point to the maximization of laser power for improved NV^–^ concentration and lifetime whenever possible.

In conclusion, we studied the effects of laser power on charge-state photoconversion in ensembles of shallow NV centers in flat and nanostructured diamonds. Surface effects favor the neutral charge state (NV^0^) over the negative state (NV^–^). This problem is exacerbated in the nanostructured samples. We show that high-power laser irradiation promotes NV^0^ to NV^–^ photoconversion, thus significantly increasing the relative abundance of NV^–^s in flat and in a nanostructured diamond. Interestingly, we find that the excess NV^–^ population is stable over timescales of tens or hundreds of milliseconds. This may provide an important advantage for magnetometry and polarization-transfer applications, where large ensembles of shallow NV^–^ are needed.

## Supporting Information Available

Charge state fluorescence ratio and filtering; effect of the readout pulse; excited state saturation; and calculation of the charged state ratio (PDF)
