# Supplementary material for: Long-Lived Ensembles of Shallow NV– Centers in Flat and Nanostructured Diamonds by Photoconversion
Source: ACS Appl Mater Interfaces. 2021 Sep 1;13(36):43221–32. doi: 10.1021/acsami.1c09825 (PMC8447188; doi:10.1021/acsami.1c09825)
Supplement: Supplementary file 1 — am1c09825_si_001.pdf [file am1c09825_si_001.pdf]

Supporting Information

Long-lived ensembles of shallow NV⁻ centers in flat and nanostructured diamond by photoconversion

Federico Gorrini[1,2], Carla Dorigoni[3], Domingo Olivares-Postigo[2,3,6], Rakshyakar Giri[3], Pietro Aprà[4,5], Federico Picollo[4,5] and Angelo Bifone[1,2,6]*

[1]*Istituto Italiano di Tecnologia, Center for Sustainable Future Technologies, via Livorno 60, 10144 Torino, Italy*
[2]*University of Torino, Molecular Biology Center, via Nizza 52, 10126, Torino, Italy*
[3]*Istituto Italiano di Tecnologia, Center for Neuroscience and Cognitive System, corso Bettini 31, 38068, Rovereto (Tn), Italy*
[4]*University of Torino, Department of Physics and "NIS inter-departmental centre", Via Pietro Giuria, 1, 10125 Torino, Italy*
[5]*National Institute of Nuclear Physics, Section of Torino, Torino 10125, Italy*
[6]*University of Torino, Department of Molecular Biotechnology and Health Sciences, via Nizza 52, 10126, Torino, Italy*
* Corresponding author. Email: federico.gorrini@iit.it.

S1 – Charge state fluorescence ratio and filtering

We collected fluorescence signals with two set-ups: a Raman confocal microscope equipped with a spectroscope, able to resolve the spectral emission from NV centers, and a home-built microscope with a photon counter module as a detector. With the Raman setup, FL was collected after exciting with a green laser at three different laser intensities (0.41 mW/μm^2, 4.1 mW/μm^2 and 41 mW/μm^2). The non-normalized spectra taken from the nanostructured sample N1 are plotted in Figure S.1a. The FL was then deconvolved into the NV^0 and NV^- individual components (green and orange curves) starting from NV^0 and NV^- FL curves available in the literature and then using the LM algorithm. Notably, the sum of the two components reproduces accurately the experimental spectra. NV curves were then integrated and summed to calculate the integrated FL of Fig. 2d. Conversely, their ratio gave the quantity labeled as $A(NV^-)/A(NV^0)$ in Fig. 2d (proportional to the charge-state ratio R discussed in the main text). The green and orange numbers in Fig. S.1a indicate the fraction of the FL emitted by NV^0 and NV^- centers, respectively. We notice that in the case considered $A(NV^-)/A(NV^0)$ increases with laser power from 0.41/0.59=0.69 to 0.58/0.42=1.38.

In the home built microscope, FL was filtered with a combination of bandpass filters, the 550-600nm and the 750nm+ (dark blue and light blue curves in Fig. S.1b, respectively), with peak transmittance of ≈85%, indicatively. The photon counter module was not able to spectrally resolve the emitted FL, and produced an integrated signal. In Fig. S.1b we overlap the emitted FL (with individual NV^0 and NV^- components) to the transmittance of the two spectral windows: the 550-600nm filter combination selects almost exclusively the NV^0 signal, but the 750nm+ filtering also contains a small NV^0-related component. Thus, the emission from NV^0 centers cannot be completely removed by spectral filtering and it can affect the NV^- signal by providing an undesirable background signal (compare Eq.3). However, at high laser power the NV^-/NV^0 ratio increases (Fig.2) and the leakage becomes less relevant.

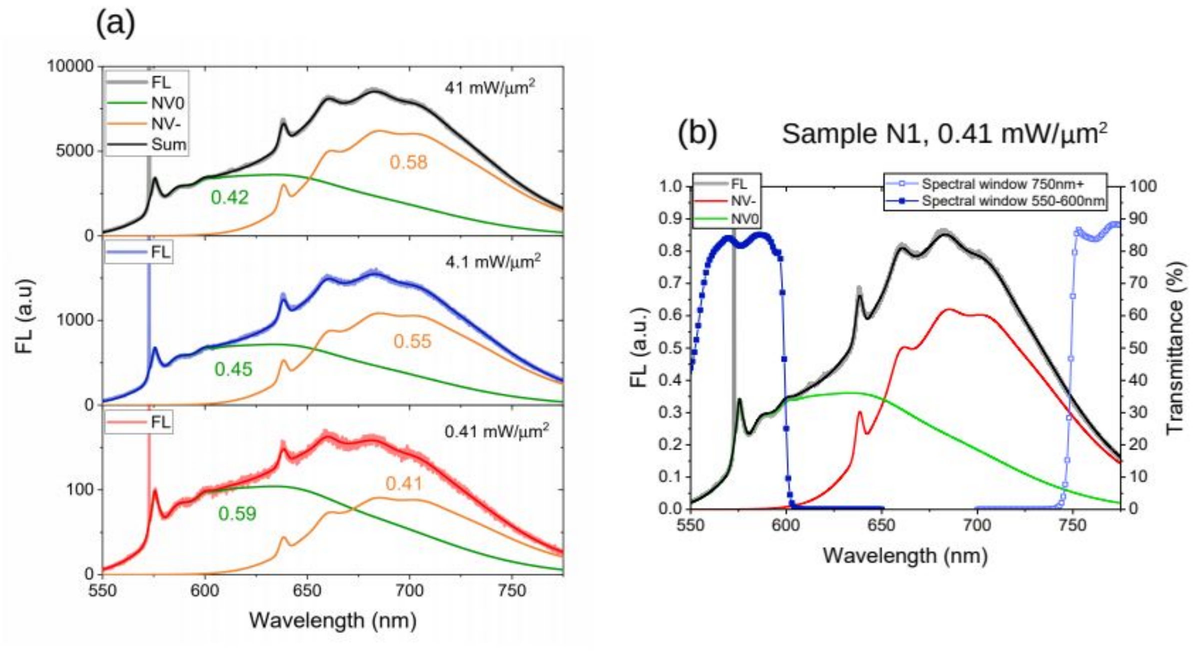

Figure S.1

S2 – Effect of the readout pulse

In most of the experiments, a readout pulse of 5 µs was adopted. We considered the possibility that even a 5 µs readout pulse may induce photoconversion, especially at high laser power. In Figure S.2 we plot the evolution of the FL signal in the dark, describing the spin and charge dynamics of sample F1 (i.e., no magnetic field applied, 750nm+ filter, analogous to the red solid curve of Fig.5a) at the highest laser power of 1.5 W, with “short” and “long” readout pulses of 0.5 µs (green curve) and 5 µs (purple curve), respectively. We did not observe substantial difference between the two curves. However, the acquisition time was affected by the length of the readout pulse (since the photon-shot noise for a Poissonian process scales as the inverse square root of the number of photons detected, or, equivalently, as the inverse square root of the acquisition time). By way of example, the green curve was acquired after 220 repetitions, while the purple curve only needed 50 repetitions (results for sample F1). We conclude that a 5 µs readout pulse ensures a good sensitivity without substantially perturbing the distribution of NV charge states even at the highest laser power.

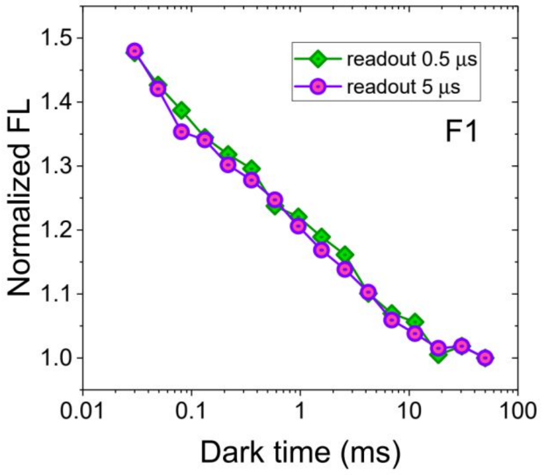

Figure S.2

S3 – Excited state saturation

Our set-up is designed to work with ensembles of NV centers, with areal density of $\approx 10^3$ μm^{-2}. Thus, several thousands of NV centers can be simultaneously excited in the laser spot. The saturation of the excited state was investigated under these conditions. We applied a continuous laser excitation and recorded the FL emission (in terms of millions of counts per second, "MC/s"). A strong magnetic field (>750 G) was also applied to suppress the effects of spin on the emitted FL. Additionally, the FL was recorded a few seconds after setting the laser power, in order to let charge dynamics reach equilibrium. Such FL values are plotted in Fig. S.3, for 550-600nm and 750nm+ spectral filtering (green and pink dots, respectively). Typically, such curves are fitted by a function of the type

$$I(P) = I_0 \frac{P}{P + P_0} + I_{off} \frac{P}{P_0} \qquad \text{(Eq. S.1)}$$

where P is the laser power and I the intensity of the FL. The first term in Eq. S.1 is the real saturation function, with P_0 being the saturation power and I_0 a scaling coefficient. The second term gives a linear offset to be subtracted. A reliable calculation of P_0 is a difficult task; we estimated it to be 2.3 W for NV^0 and 8.5 W of NV^- (corresponding to a saturation intensity of 230 mW/μm^2 and 850 mW/μm^2, respectively, for a laser spot size of 10 µm).

Again, we see that nanostructured and flat samples differ in the values and in the shapes of the two curves. The nanostructured samples do not display an onset of saturation in either curves, while for the flat samples the NV^0 curves start to flex. We notice here that the effective laser intensity for the nanostructured samples might be lower by a factor of ≈ 3 compared to the flat samples, because of irradiation on tilted surfaces. Even by rescaling the laser power by the corresponding factor, the flat and nanostructured samples behave differently. Ultimately, this indicates that the laser power must be further increased to approach saturation in the nanostructured samples.

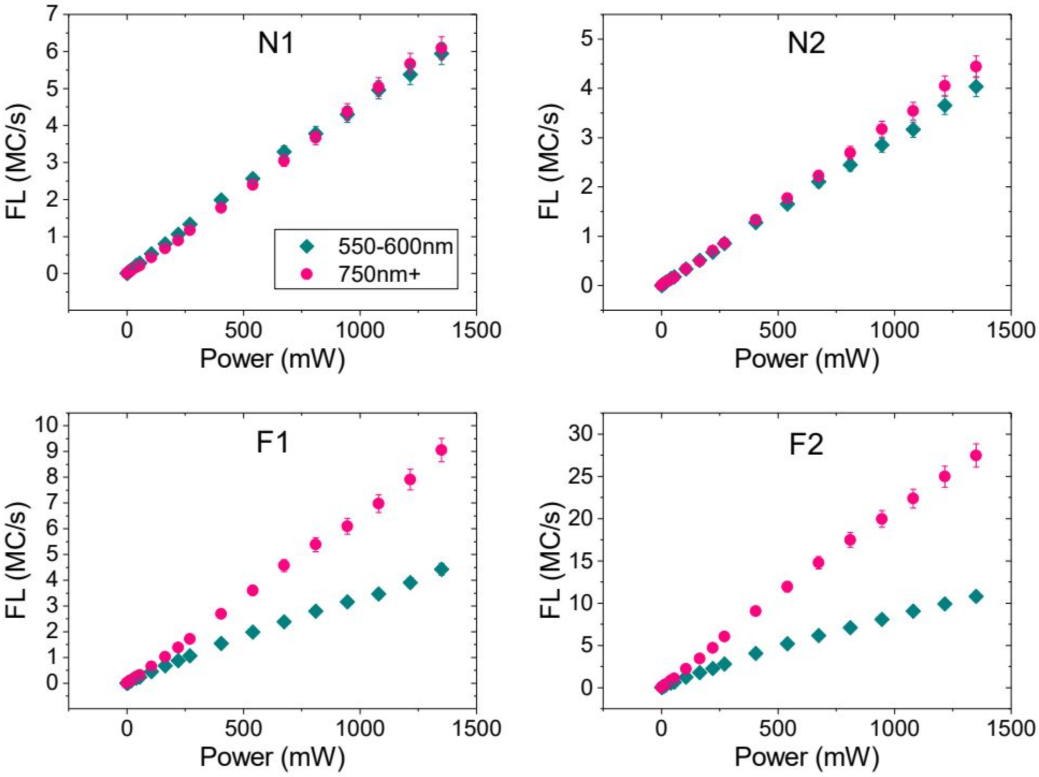

Figure S.3

S4 – Calculation of the charged state ratio

An important parameter that characterizes the samples is the ratio between the concentrations of negatively centers to neutral centers, defined as R=[NV$^-$]/[NV^0]. A simple pulse sequence (Fig.3c in the main text) makes it possible to estimate this ratio. To this end, a strong magnetic field (750 G) is applied along the [100] direction to suppress the spin polarization mechanism, and selectively investigate charge dynamics. Indeed, a sufficiently strong magnetic field (>600 G) mixes the spin states and quenches the mechanism responsible for the m_s=0 polarization, leading to a reduction of the spin-related component in the fluorescence. We then record fluorescence through two spectral windows, the 550-600nm (for the NV^{0}s) and the 750nm+ (for the NV$^-$s), as shown in section S1 of the supplementary material. The two signals are fitted by stretched exponentials.

Only NV^0 signal passes through the 550-600nm filter. The time dependence of the FL is given by

$$I^0(t) = I^0{}_{eq}\left[1-\delta_0 e^{-\left(t/T_r\right)^n}\right] \qquad \text{(Eq. S.2)}$$

On the contrary, a fraction of the NV^0 fluorescence leaks through the 750nm+ window. The composite signal now has two components:

$$I(t) = kI_{eq}^{0}\left[1-\delta_0 e^{-\left(t/T_r\right)^n}\right] + I_{eq}^{-}\left[1+\delta_{-} e^{-\left(t/T_r\right)^n}\right] \qquad \text{(Eq. S.3)}$$

Here, I_{eq}^{-} and I_{eq}^{0} are the levels of fluorescence at equilibrium, T_r and n govern the dynamics of charge conversion, and δ_0, δ_-, are positive parameters that indicate the initial out-of-equilibrium level of fluorescence. The number k quantifies the amount of NV^0 leakage, and it can be estimated from the FL profile and the spectral windows transmittivity (here k≈0.19). From Eq. S.2 and knowing k, it is possible to recover the pure NV^- signal in Eq. S.3.

We now suppose that the total number of NV centers in the neutral or in the negative charged state is not affected by the laser pulses (*i.e.* we do not consider the potential contribution of NV^+, which are only present in very low concentration). Thus, a reduction in $N^-(t)$, the number of NV^-s, is counterbalanced by a gain in $N^0(t)$, the number of NV^0s, such that $N^-(t) + N^0(t) = N_{tot} = cnst$. With this assumption, the fractions of NV^- and NV^0 centers over the total at equilibrium are $\frac{N_{eq}^-}{N_{tot}} = \frac{\delta_0}{\delta_- + \delta_0}$ and $\frac{N^0{}_{eq}}{N_{tot}} = \frac{\delta_-}{\delta_- + \delta_0}$. The charge state ratio is then given by $R = \frac{N_{eq}^-}{N_{eq}^0} = \frac{\delta_0}{\delta_-}$. We notice that the charge state ratio R is different from the ratio between the individual defects fluorescence. In fact, the two centers have different absorption cross sections ($\sigma^- = 3.1\text{x}10^{-17}$ cm^2 [51], $\sigma^0 = 1.8\text{x}10^{-17}$ cm^2 [52]) and relaxation times (τ^-=12 ns, τ^0=21 ns [53]), and the fluorescence ratio should be scaled by a factor of ≈ $\frac{f^0\sigma^0\tau^-}{f^-\sigma^-\tau^0}$, where f^- and f^0 depend on the detection condition (they are the fractions of the total emitted FL collected through the combination of filters in use). Any further comparison between FL ratio of section S1 and the process here illustrated is complicated, since the full FL spectra (of Fig. 2 and Fig. S.1) were acquired with no strong magnetic field applied and they also contain a spin-related component.

As an example, this procedure is illustrated in Figure S.4 for two samples (flat F2 and nanostructured N2) at the high power of 1500 mW. The magnetic field of 750 G reduced the spin polarization and only leaves the part of charge dynamics (from red solid points to red empty points in panels a and b). The NV^0-related FL is not affected by the presence of a magnetic field (blue curves). We see that for sample N2 the initial values of NV^- are higher. This is not surprising, as the initial contrast depends mostly on the photoconverted charges. For sample N2, the NV^- concentration is so low that a laser can affect dramatically the charge states, resulting in a high initial value of the curve. However, the difference between the two red curves (solid and empty points) is larger in sample F2, indicating a better spin polarization overall. We also note that the slope of the red curves is different between samples F2 and N2, indicating a faster $NV^- \rightarrow NV^0$ charge recombination in the dark for the nanostructured sample (Fig S.4b). The differences between samples F2 and N2 are illustrated also in Fig. S.4c and S.4d, where we applied the above-mentioned analysis to calculate the NV^- and NV^0 population fractions, and the charge state ratio R under a strong magnetic field. In sample F2, the fraction of NV^- remain high even after 100 ms (53% of the total NVs, red curve), while it decays to 10% (rapidly, right after 10 ms) in sample N2.

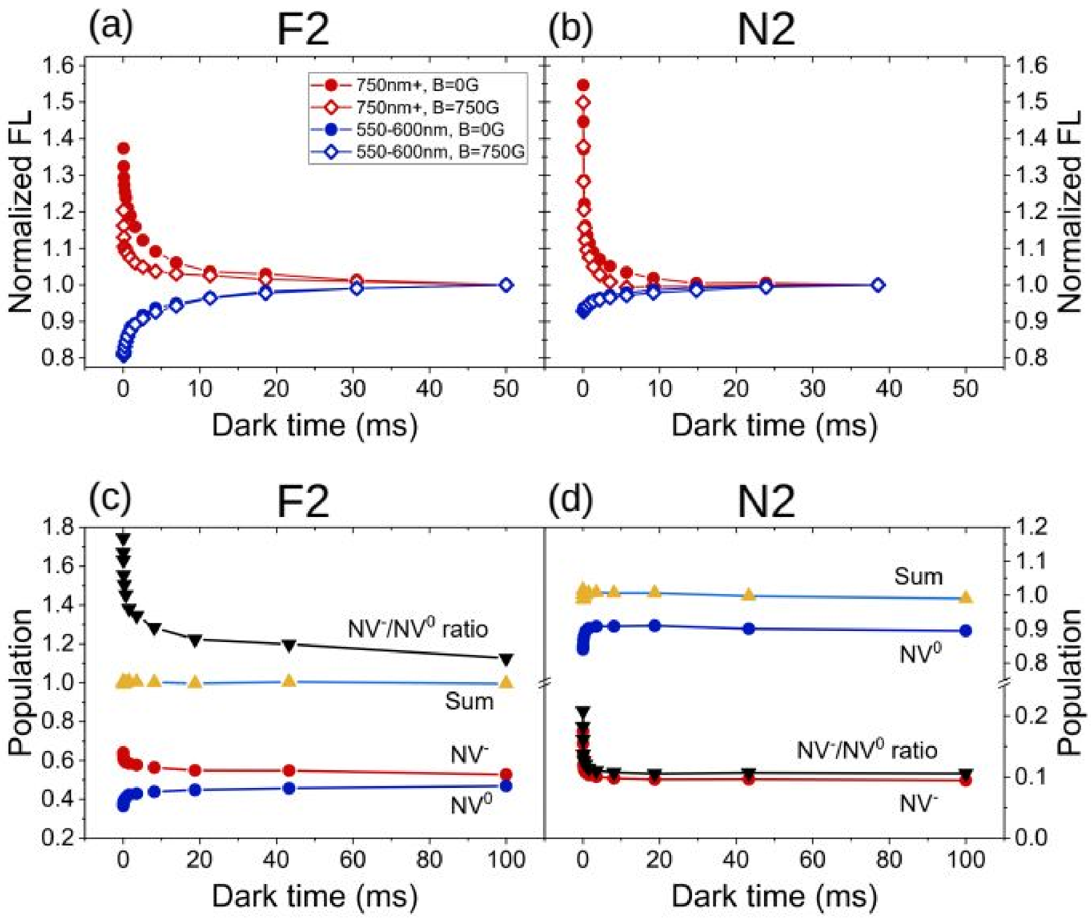
(a)
F2
(b)
N2
750nm+, B=0G
750nm+, B=750G
550-600nm, B=0G
550-600nm, B=750G
Normalized FL
Dark time (ms)
(c)
F2
(d)
N2
Population
NV⁻/NV⁰ ratio
Sum
NV⁻
NV⁰
Dark time (ms)

Figure S.4
